# Characteristics of respiratory microdroplet nuclei on common substrates

**DOI:** 10.1098/rsfs.2021.0044

**Published:** 2021-12-10

**Authors:** Alexandros Kosmidis-Papadimitriou, Shaojun Qi, Ophelie Squillace, Nicole Rosik, Mark Bale, Peter J. Fryer, Zhenyu J. Zhang

**Affiliations:** ^1^ School of Chemical Engineering, University of Birmingham, Edgbaston, Birmingham B15 2TT, UK; ^2^ DoDxAct Ltd, Wells BA5 2LW, UK

**Keywords:** respiratory, microdroplet, nuclei, substrates, transmission, virus

## Abstract

To evaluate the role of common substrates in the transmission of respiratory viruses, in particular SARS-CoV-2, uniformly distributed microdroplets (approx. 10 µm diameter) of artificial saliva were generated using an advanced inkjet printing technology to replicate the aerosol droplets and subsequently deposited on five substrates, including glass, polytetrafluoroethylene, stainless steel, acrylonitrile butadiene styrene and melamine. The droplets were found to evaporate within a short timeframe (less than 3 s), which is consistent with previous reports concerning the drying kinetics of picolitre droplets. Using fluorescence microscopy and atomic force microscopy, we found that the surface deposited microdroplet nuclei present two distinctive morphological features as the result of their drying mode, which is controlled by both interfacial energy and surface roughness. Nanomechanical measurements confirm that the nuclei deposited on all substrates possess similar surface adhesion (approx. 20 nN) and Young's modulus (approx. 4 MPa), supporting the proposed core–shell structure of the nuclei. We suggest that appropriate antiviral surface strategies, e.g. functionalization, chemical deposition, could be developed to modulate the evaporation process of microdroplet nuclei and subsequently mitigate the possible surface viability and transmissibility of respiratory virus.

## 1. Introduction

Transmission of respiratory viruses can take place in different modes, either directly via contact between individuals, indirectly via commonly touched objects or surfaces, or directly through the air in the form of large droplets or small aerosols [1]. Surface transmission, in particularly via surface fomite, was viewed as one of the primary concerns since the initial stages of the pandemic caused by severe acute respiratory syndrome coronavirus-2 (SARS-CoV-2) in 2020 [2]. One of the early studies suggested that SARS-CoV-2 remains viable in aerosols for at least 3 h, and that SARS-CoV-2 is more stable on plastic and stainless steel (SS) than on copper and cardboard [3], which highlighted the unique role of common surfaces in virus transmission during the pandemic. The impact of substrate on the surface viability of virus was further demonstrated with a large range of Middle East respiratory syndrome coronavirus (MERS-CoV) [4].

There have been compelling arguments that the transmission of SARS-CoV-2 after touching surfaces should be considered as relatively minimal [5], given that it is improbable that an infected person coughs or sneezes on a surface (with sufficient quantity of infectious virus), and someone else touches that surface shortly after (within 1–2 h) [6]. This rationale is sound and sensible, on the assumption that surface transmission takes place via a large quantity of respiratory fluid and that the virus would be inactivated beyond the timeframe suggested. An extensive list of evidence, including superspreading events, long-range transmission, asymptomatic transmission, was given to support that the dominating transmission route of SARS-CoV-2 is airborne [7,8]. Although the likelihood of surface transmission is no longer as significant as perceived at the initial stage of the pandemic, the possible presence of infectious virus on solid substrates, in particular on high-touch environmental surfaces, could have significant implications for both social and healthcare practice.

There remain significant knowledge gaps in drawing conclusions on the possible role of surface in preserving and transmitting SARS-CoV-2 due to the multitude of complexities involved. This is highlighted in a systematic review by Onakpoya and colleagues focusing on the role of fomite transmission over 64 studies [9], which concluded that no evidence is available to confirm viral infectivity or transmissibility via fomites, but that none of the studies surveyed is sufficiently methodologically robust to adequately address the question. A similar reflection questioning the unlikelihood of indirect transmission through contaminated surfaces has been reported recently [10]. A core element that underpins the inconsistent viewpoints on the transmission pathways of SARS-CoV-2 is the physico-chemical properties of the microdroplets. Other than the discrepancy over the definition of aerosols, droplets, particles and droplet nuclei perceived by researchers from different disciplines [11], diameters of the exhaled droplets can cover a broad range, from 0.1 to 1000 µm [12,13], for which the fluid mechanics and evaporation kinetics vary substantially. Literature suggests that sneezing may generate droplets between 0.5 and 12 µm in diameter whereas breathing and speaking may result in droplets with at an averaged diameter of 1 µm [14–17].

It is therefore crucial to further understand the characteristics of aerosol droplets once they are deposited on common surfaces. Evaporation kinetics of microlitre and picolitre droplets of pure liquid, e.g. water or organic solvents, have been extensively studied in the past [18,19]. However, the complex compositional nature of saliva, consisting of salts, proteins and lipids could influence not only the evaporation process of the microdroplet, but also the location of the viruses in the final nuclei, and consequently their viability and transmissibility on solid substrates [20,21]. Previous studies in which model respiratory liquids were used reported that the generated microdroplets would crystallize on the substrate as the result of their evaporation process [22,23]. It is unclear whether the surface crystallization, as part of the drying process, could inactivate the viruses because they are enveloped in the crystals, or the crystals actually preserve the viruses and prolong their viability upon rehydration. Furthermore, characteristics of the microdroplet nuclei could have a direct impact on the effectiveness of sample collection protocols in that the dissolution kinetics of the crystalline phase is much slower than the amorphous phase [24]. Finally, adhesion of microdroplet nuclei to the underlying substrate could be critical to contact transmission. Literature suggests that the molecules contained in human saliva, such as salivary agglutinin [25], salivary proteins MUC5B, cysteine-rich glycoprotein 340 [26] and secretory leucocyte protease inhibitor [27], could introduce inhibitory and antiviral effects. However, artificial saliva that consisted of mucin and inorganic electrolytes was used in the present work due to health and safety restrictions.

In the present work, we investigated systematically the morphological and nanomechanical properties of microdroplet nuclei and their formation kinetics as a function of the surface in contact. Using an advanced inkjet printing set-up, we were able to generate picolitre droplets and subsequently deposit them onto five inanimate materials that are commonly found in daily life, replicating the surface transmission route of viruses. We found that surface characteristics, including both surface energy and roughness, could influence the evaporation process of microdroplets, the structure of the resulting nuclei, and consequently the viability and transmissibility of virus.

## 2. Methodology

### 2.1. Materials

Phosphate-buffered saline, mucin extracted from porcine stomach (M1778), magnesium chloride, calcium chloride, ammonium chloride and Dulbecco's modified Eagle's medium (DMEM) were purchased from Sigma-Aldrich (Somerset, UK).

### 2.2. Artificial saliva

Artificial saliva, an aqueous mixture of salts, nutrients and mucin, was prepared to replicate human saliva [28]. The addition of inorganic ions in the mixture offers osmotic balance and buffering especially during the injection process [28–30]. The artificial saliva composition used in the present work is shown in table 1 [17,31–33].

**Table 1.** Composition of artificial saliva per litre of aqueous solution in deionized water.

compound	quantity	compound	quantity
MgCl_2_·7H_2_O	0.04 g	Na_2_HPO_4_	0.42 g
CaCl_2_·H_2_O	0.13 g	(NH_2_)_2_CO	0.12 g
NH_4_Cl	0.11 g	mucin	1.00 g
KCl	1.04 g	KH_2_PO_4_	0.21 g
DMEM	1 ml	NaCl	0.88 g

### 2.3. Picolitre droplet generation

A customized Jetxpert Print Station (Imagexpert, NH) was used to generate arrays of picolitre droplets of the artificial saliva that were deposited onto various solid substrates. The print station uses a conveyor belt instead of a single linear stage to improve processing efficiency. The print head (GH2220, Ricoh, UK) was thoroughly cleaned with distilled water, followed by the prepared artificial saliva, prior to the droplet generation. To prevent any potential clogging of the print head by the drying saliva solution, an automated printing cycle that ejects 200 droplets every 5 s was programmed to keep the nozzles wet and prevent the accumulation of mucin.

To generate the desired droplet size, the print head driving signals were optimized in terms of voltage amplitude and pulse timing. The Jetxpert print station was equipped with a strobing camera and the relevant software to measure the droplet size. Once droplets with a diameter of approximately 10 µm were produced consistently, the print head was placed over the conveyor stage to generate arrays of droplets that were approximately 175 µm apart by printing with a resolution of 300 × 300 dpi. A standard USB camera (Hayear 1136) was mounted above the printing stage to record videos (30 fps) of the printed artificial saliva droplets and to monitor the averaged drying time on each substrate. Five solid substrates of distinctive characteristics were used to represent the common surfaces: borosilicate glass slides to represent glass and ceramics, polytetrafluoroethylene (PTFE) to represent non-stick coatings, SS that is commonly used for door handles and hand rails, acrylonitrile butadiene styrene (ABS) that has a wide application in household and consumer goods, and melamine that is used for dinnerware and laminate flooring.

### 2.4. Fluorescence microscopy

Fluorescence microscopy was carried out to identify the residues of the deposited microdroplets. Alexa Fluor 488 Maleimide of excitation/emission wavelength 493/516 nm was used for fluorescence imaging purposes. For one dedicated set of experiments, the fluorophore was dissolved in dimethyl sulfoxide at a concentration of 1 mM, of which 10 µl were introduced to 10 ml of artificial saliva solution prior to the printing process. Sample images were captured after 5 days of drying, using an inverted fluorescence microscope (Olympus IX71).

### 2.5. Contact angle goniometer

An optical tensiometer (Theta Flow, Biolin Scientific, UK) was used to measure both advancing and receding contact angles (CAs) of artificial saliva on all five solid substrates, for which droplet of 4 µl was produced by the associated micro-syringe.

To quantify the surface free energy (SFE) of these substrates, 2 µl diiodomethane (apolar) or water (polar) were placed on the substrates, and equilibrium CAs were recorded subsequently. Owens/Wendt theory describes the surface energy of a solid as having two components, a dispersive component and a non-dispersive (or polar) component. Mathematically, the theory is based on the combination of two fundamental equations (Good's equation and Young's equation) that describe interactions between solid surfaces and liquids [34,35]. This equation has a linear form *y* = mx + *b*, wherein:

2.1 $$y=\;\frac{\sigma_{L}.(\cos\theta +1)}{2\;.\;\sigma {{\;}_{L}^{D}}^{1/2}};x=\frac{\sigma {{\;}_{L}^{P}}^{1/2}}{\;\sigma {{\;}_{L}^{D}}^{1/2}};m=\;\sigma_{S}^{P^{1/2}};b=\sigma_{S}^{D^{1/2}}.$$

Diiodomethane has a relatively high overall surface tension of 50.8 mN m^−1^ but no polar component, so that $\sigma_{L}=\sigma_{L}^{D}=$ $50.8\;\mathrm{mN}\;m^{-1}$, while water has a surface tension $\sigma_{L}^{P}=46.4\;\mathrm{mN}\;m^{-1}$ for the polar component and $\sigma_{L}^{D}=26.4\;\mathrm{mN}\;m^{-1}$ for the disperse component. The slope *m* of that line is used to calculate the polar component of the surface energy of the solid $\sigma_{S}^{P}$ and the intercept *b* is used to calculate the dispersive component of the surface energy of the solid $\sigma_{S}^{D}$, the overall SFE of the solid being defined as $\mathrm{SFE}=\sigma_{S}^{D}+\sigma_{S}^{P}.$

### 2.6. Atomic force microscopy

Surface topography and characteristics of the dried droplets were examined using an atomic force microscope (Dimension 3100, Bruker). All imaging and force curve requisition was performed with silicon nitride cantilevers (PNP-TR-Au, spring constant 0.08 N m^−1^, Apex Probes Ltd, UK). All cantilevers were subject to the same cleaning routine (rinsing with ethanol followed by exposure to UV-ozone for 15 min) before the first use and between every two samples. Force measurements on the dried artificial saliva nuclei were acquired with a maximum loading force of 5 nN. At least 100 repetitions, in the form of a 7 × 7 matrix (200 nm apart in both *x* and *y* directions), were acquired at 2–3 random locations within the area of interest. Young's moduli of the dried products were estimated by fitting the approaching component of the force curves with Sneddon model (conical indenters):

2.2 $$F=\frac{E}{1-v^{2}}\cdot \frac{2\tan\alpha }{\pi }\cdot \delta^{2},$$

where *E* and *ν* are the Young's modulus and the Poisson's ratio (0.5 for organic compound) of the materials being indented, respectively; *α* is the semi-opening angle of the atomic force microscope (AFM) tip which is 35° in the present study; *δ* is the indentation depth which is calculated by subtracting the cantilever deflection from the measured sample height.

## 3. Results and discussion

### 3.1. Microdroplet generation

**Figure 1.**
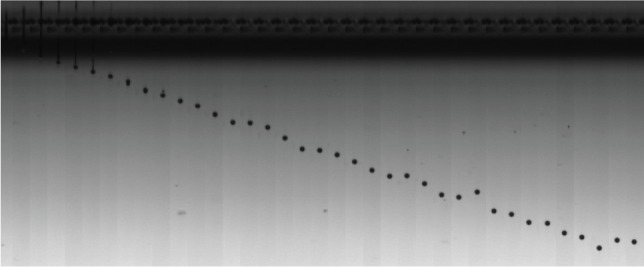
Mosaic of images captured from 37 separate ejection events by the Jetxpert camera to show the flight trajectory of a single artificial saliva droplet from the moment of ejection (far left). Diameter of the droplet is 10 µm.

The Ricoh GH2220 print head deployed in the present work was chosen due to its highly adaptable performance with different types of fluid. Although the viscosity of the artificial saliva was well below the ideal operating point for the print head, there is a wide latitude to modify the print head driving signals. To measure and adjust droplet size, the camera of the Jetxpert drop watcher is triggered in synchronization with the GH2220 drive electronics (Meteor Inkjet Ltd, UK). This is a standard method to capture the flight trajectory of individual droplets as they are ejected from the inkjet head. The goal was to find the optimal pulse shape and voltage amplitude to actuate the piezoelectric elements of the print head, so as to eliminate satellite droplets present and reach the desired droplet size. Figure 1 presents a series of images captured, showing the trajectory of a single artificial saliva droplet of approximately 5 pl volume being ejected from the print head. We were able to capture the entire process, from the moment when the droplet was detached from the nozzle (far left) and the subsequent movement in the air without any satellite drops.

The two rows of nozzles of the GH2220 enabled printing in a single pass with a pixel addressability of 300 dpi, which could produce droplets as close together as 85 µm. To minimize the possibility of any uncontrolled merging of adjacent drops across the range of surfaces studied, a print image using an ordered dither of approximately 23% coverage was implemented, resulting an average droplet separation of approximately 175 µm. With these settings, and belt speed of just 5 m min^−1^, the print frequency was kept low (less than 1 kHz), thus minimizing the chance of nozzle failure due to the sub-optimal rheology. A camera was positioned next to the solid substrate to capture the drying of the deposited droplets from the point the conveyor came to rest, approximately 1 s after printing.

The ability to consistently and rigorously generate microdroplets of uniform size provides a substantial advantage over the other techniques in generating aerosol droplets such as atomizer and nebulizer. It offers the assurance to compare droplet nuclei formed on different substrates, with minimal variation in the droplet characteristics.

### 3.2. Evaporation of the deposited droplet

**Figure 2.**
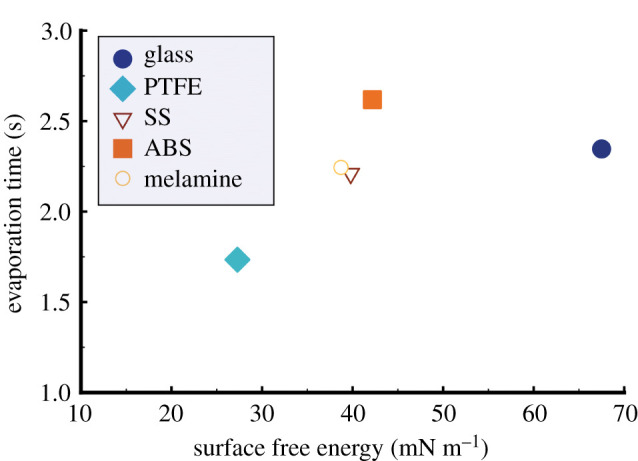
Evaporation time of artificial saliva microdroplet on five different substrates as a function of their corresponding SFE that was calculated using diiodomethane and deionized water. Error bars are of similar size to the dimension of the symbols used, and hence not shown.

Droplets of 10 µm diameter (volume is approximately 4.8 pl) were deposited on five different surfaces, namely: glass, PTFE, SS, ABS and melamine. The time it took for the droplet to evaporate on the substrates, upon deposition, was estimated using the videos captured and is presented (figure 2) as a function of the corresponding SFE established based on deionized water and diiodomethane. The drying time estimated appears to correlate with the SFE of the substrates: the greater the SFE is, the longer it takes for the droplet to evaporate.

Evaporation kinetics, including time, of microlitre liquid droplets on solid substrate have been studied extensively at millimetre scale [18,19]. It is widely accepted that there are two limiting drying scenarios: (i) the contact line of the liquid/solid interface remains constant throughout the drying while CA decreases, called constant contact radius (CCR) mode and (ii) droplet radius reduces while the CA remains the same, called constant contact angle (CCA) mode, which is often observed on hydrophobic substrates [36]. It is, however, worth noting that variations in both the physical and chemical characteristics of the substrate could help to enhance contact line pinning, although strongly hydrophobic substrates could be exceptions [37].

For droplets of picolitre volume, Talbot *et al*. [38] investigated the evaporation kinetics of water and ethanol droplets on surfaces with different wettability and thermal conductivity. The droplet volume studied ranges from 4 to 65 pl, and the drying time for water droplets was around 4 s for the surfaces used. This is in a very similar time frame to that observed in the present work, which is not surprising because the artificial saliva used in our work consists of 97% water. It was not possible to measure the CA of the picolitre droplet in the present investigation, but previous studies suggest that it would be several degrees less than that measured using microlitre droplet [38]. Although drying and evaporation kinetics of picolitre droplets are beyond the scope of this work, the observed correlation between drying time and SFE does not seem to agree with the previous finding that evaporation on hydrophilic substrates is faster than on hydrophobic substrates. This inconsistency could be attributed to the substrates used, or the surface adsorption of mucin molecules upon deposition. We highlight that the variations in the actual drying time of artificial saliva microdroplets of 10 μm are within 2 s (figure 2), which indicates that transfer of aerosol droplet in liquid state is unlikely, and suggests that the effect of surface hydrophobicity on the drying time of microdroplets (less than 10 µm) is not a significant factor in the context of surface transmission. The short time (a few seconds) for these biological picolitre droplets to reach full dryness, as opposed to the length scale (minutes to tens of minutes) it takes for micro- to macro-sized droplets, underlines the importance of focusing on the physico-chemical and virological characteristic of the dried nuclei of such, rather than solely on fresh respiratory deposition in terms of surface transmission.

### 3.3. Advancing and receding contact angle measurements

**Figure 3.**
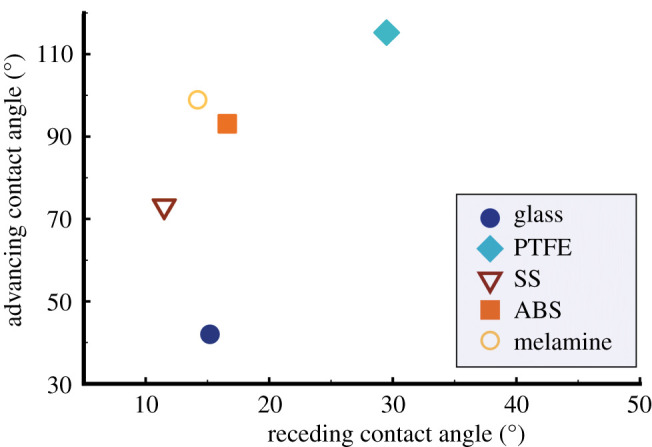
Advancing CA of artificial saliva on the five substrates as a function of the corresponding receding CA.

Advancing and receding CAs of artificial saliva (2 µl) were measured on glass, SS, PTFE, ABS and melamine, as presented in figure 3. Of the five substrates investigated, glass shows the smallest advancing CA (42.0°) and the second smallest receding CA (15.2°) of the range, while PTFE results in the greatest ones (115.2° and 29.5° respectively), which correlates with their SFE values: 67.5 mN m^−1^ and 27.3 mN m^−1^ for glass and PTFE, respectively. SS, ABS and melamine present intermediate values, in terms of SFE and CA of artificial saliva, showing a similar correlation between advancing and receding CA values. The advancing CA on SS is noticeably less than that on ABS or melamine.

The differences in SFE, advancing and receding CAs between the five substrates surveyed are likely due to the synergistic effect of surface characteristics, e.g. chemical nature, topography and roughness [39,40]. Glass substrate shows the lowest roughness (*R*_a_), 1.1 ± 0.1 nm (obtained by AFM images over an area of 50 × 50 µm), followed by ABS and melamine with 3.8 ± 0.7 nm and 3.7 ± 0.8 nm respectively, while PTFE and SS present great roughness with *R*_a_ = 19.4 ± 3.3 nm and 33.6 ± 6.1 nm, respectively. Although SS presents a high surface roughness, both advancing and receding CAs on SS are less than those on ABS and melamine, which is likely attributed to the hydrophilic nature of SS, while ABS and melamine are less polar. This supports our observation of artificial saliva CAs on the solid substrates and is consistent with our previous experimental work in which SS samples of different surface finishing were investigated [41]. It is worth noting that substrates such as SS, ABS and melamine, whether smooth or rough, can present high advancing CA but low receding CA, suggesting that it is possible for those less polar substrates to have an increased interaction with the artificial saliva once the surface is already wet, while it would not have been the case when dry. At the opposite, PTFE only presents high advancing and high receding CA. The surface characteristics discussed at the macrodroplet scale, including both polarity and roughness, are still important consideration at the microdroplet scale.

### 3.4. Droplet nuclei characterization

**Figure 4.**
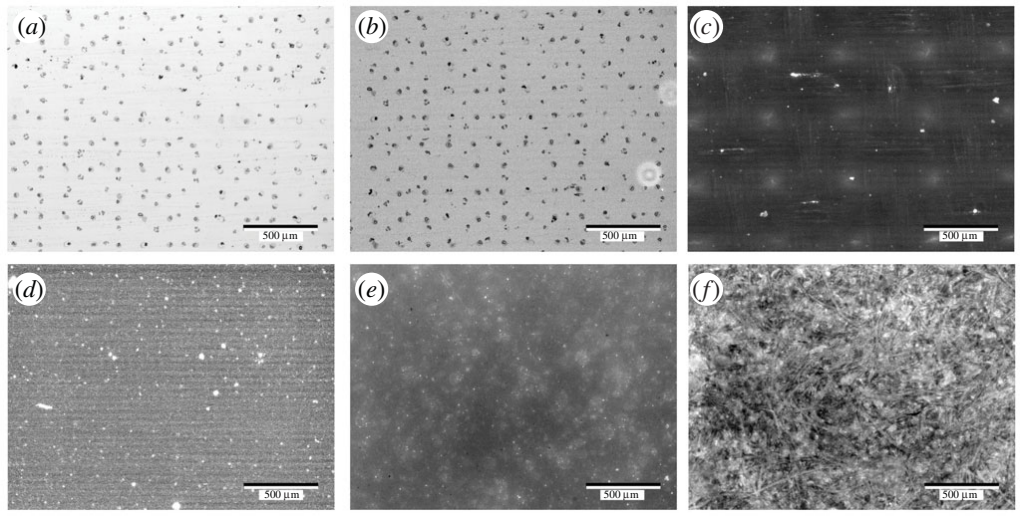
(*a*) Bright field and (*b*) fluorescence images of artificial saliva microdroplet nuclei on glass. Fluorescence images of droplet nuclei on (*c*) PTFE, (*d*) SS, (*e*) ABS and (*f*) melamine upon deposition of artificial saliva added with fluorophore (Alexa Fluor 488 Maleimide). Scale bar corresponds to 500 µm.

Morphology of the microdroplet nuclei with microscopic and nanoscopic spatial resolution was subsequently investigated using fluorescence microscopy and atomic force microscopy, respectively. Artificial saliva mixture containing Alexa Fluor 488 Maleimide was deposited on a set of substrates using the same printing conditions and imaged by a fluorescence microscope afterwards. The acquired images are shown in figure 4 where the array of droplet nuclei is distinctively identifiable on some of the substrates, in particular on the glass substrate (dark dots presented in figure 4*a,b*). The series of solid particulates confirm the robustness of the method in preparing the microdroplet nuclei. They (the bright dots) could be seen on PTFE (figure 4*c*) and SS (figure 4*d*), but are no longer noticeable on substrates such as ABS (figure 4*e*) and melamine (figure 4*f*), which is likely due to the signal fluctuation from the background.

**Figure 5.**
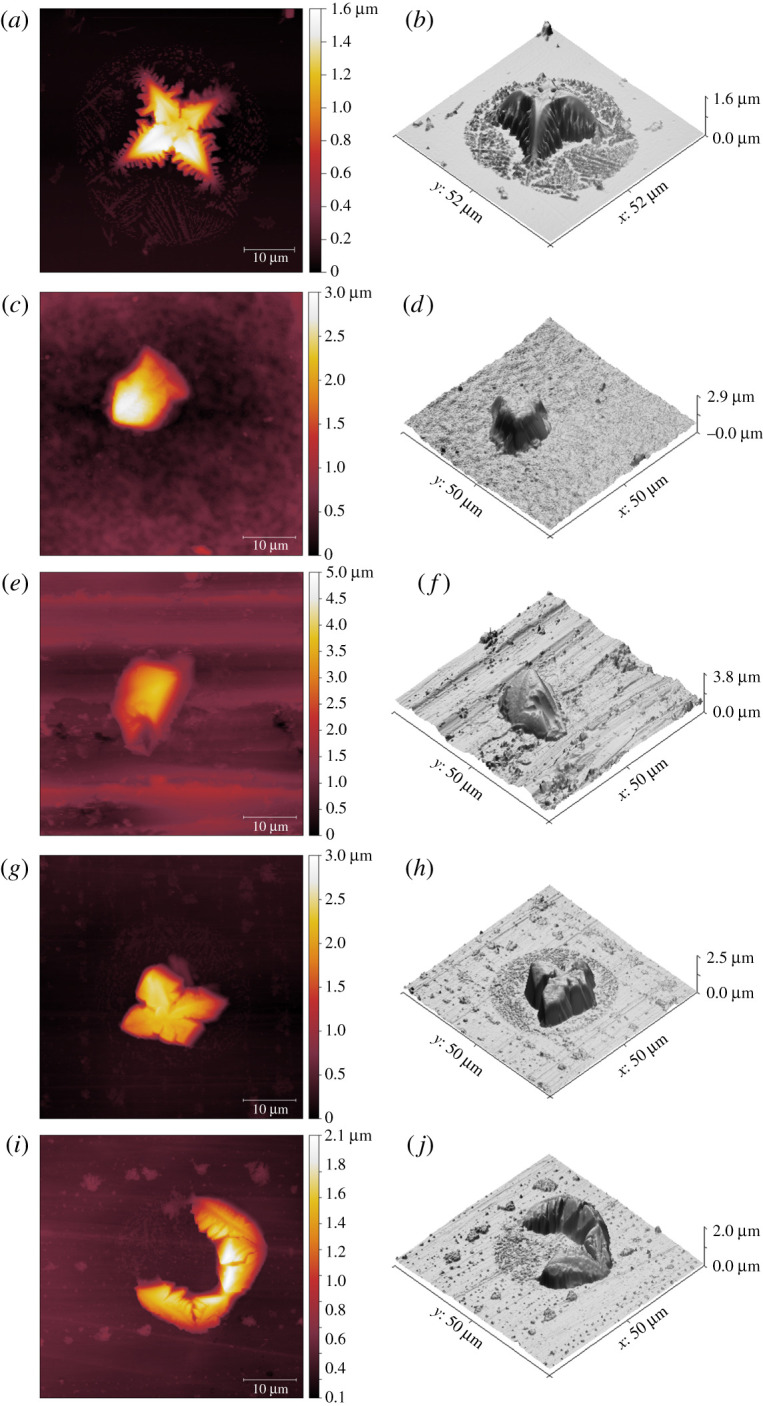
Morphology and the corresponding three-dimensional reconstructed images of artificial saliva droplet nuclei formed on (*a,b*) glass, (*c,d*) PTFE, (*e,f*) SS, (*g,h*) ABS and (*i,j*) melamine.

To establish fine details of the microdroplet nuclei, AFM was deployed to survey different regions of the solid substrates, of which representative images are shown in figure 5. Two distinctive morphological features were observed: large crystals surrounded by small solid residues (islands), as shown on glass, ABS and melamine (figure 5*b*,*h*,*j*), or large crystals without any noticeable neighbouring residue (figure 5*d,f*). As a result of the evaporation process of artificial saliva, such notable variation could be solely attributed to the characteristics of the solid substrate.

As explained in a recent study by Lieber *et al*. [42], respiratory fluid such as saliva consists of a range of inorganic salts and proteins, with a major fraction of water that will evaporate under typical ambient conditions. They used an acoustic levitator to study the temporal evolution of saliva droplets that underwent evaporation in the air and reported that the ratio between the equilibrium and initial diameter of a droplet is 20%, assuming an initial combined mass concentration of salts and proteins of 0.8%. Although this ratio is unlikely to be applicable for the droplets deposited on a substrate, their study highlights the significant difference between the evaporation characteristics of water and saliva, and suggests that both precipitation and crystallization could take place during the evaporation of saliva droplets.

Concerning the drying process of a surface deposited macroscopic droplet, a capillary flow induced by the evaporation of water molecules at the air/liquid interface generates a convective mass transfer phenomenon and liquid at the ridge is replenished by liquid from the interior [43]. The convective flow could carry the dispersed solutes to the solid/liquid contact line, resulting in a circle of solid deposit, known as the coffee ring effect [43]. This flow would be countered by Marangoni flows that redistribute the particles back to the centre of the droplet [44]. In the present work, no coffee ring effect was observed on any of the substrates, suggesting that either the evaporation time was not sufficient to drive the solutes, such as proteins, to the edges of the microdroplet, or the Marangoni flows were strong enough to counter the convective mass transfer. Instead, solid particles with crystallization features were seen on all substrates surveyed, which is consistent with the results reported by Vejerano & Marr [22] who studied the transformation of a mixture of mucin, salt and surfactants, 1,2-dihex- adecanoyl-sn-glycero-3-phosphocholine (DPPC), as a function of relative humidity. Using fluorescence labelled mucin and DPPC, they were able to demonstrate that the droplet exhibited an initial core–shell structure, with a great concentration of mucin at the shell (air/liquid interface). Considering the fast evaporation kinetics observed in the present work, it is very probable that mucin molecules were kept at the shell while the inorganic salts underwent the crystallization process in the centre as the water molecules evaporated.

The dimension and morphology of these droplet residues exhibit explicitly a compliance with the corresponding SFEs of the substrates on which the picolitre droplets landed and evaporated. AFM images in figure 5 suggest that, upon the initial contact with the solid substrate, the proteinaceous microdroplets had a maximal contact area on substrates with high SFE, but kept a minimal contact with the ones with low SFE. For instance, the droplet residue on glass spans a width of 35 µm, which is the largest of the five substrates, and a peak height of approximately 1.5 µm, which is the smallest among the substrates. By contrast, the droplet nuclei on PTFE measure only approximately 10 µm in diameter but are 3 µm high, which is the tallest in this range. This observation echoes the results reported previously concerning the evaporation process of picolitre droplets [38] that water droplets followed a pinned contact line on glass but a moving contact line on PTFE, which results in droplet nuclei of different morphology and crystalline phase.

**Figure 6.**
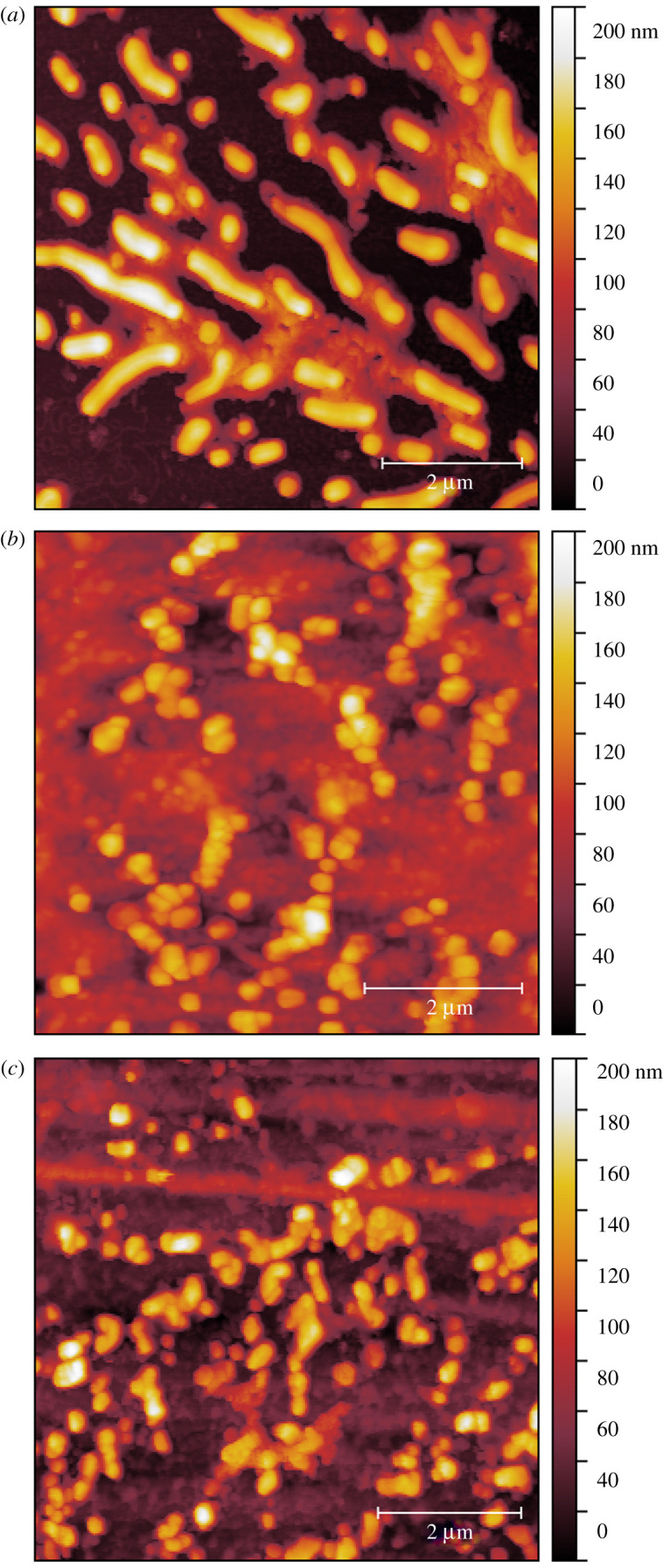
Small islands of nuclei formed aside the large crystal on (*a*) glass, (*b*) ABS and (*c*) melamine. Scale bar is 2 μm.

Glass, ABS and melamine possess minimal surface roughness and low receding CAs (figure 4), on which the residue of artificial saliva microdroplets was found in the form of small dendrites or islands across the microdroplet residue region, next to the large crystal (figure 6). This correlates with the low receding CA measured on the same substrates at the millimetre scale, which evidences that CCR mode is appropriate for the evaporation of respiratory microdroplets on substrates with high SFE. While the initial spreading of a microdroplet on the solid substrate is reflected by the advancing CA, it is the receding CA that plays a critical role in the subsequent evaporation process. Although SS possesses low receding CA (figure 4), only a large crystal was found in the droplet region, which is probably due to either its excessive roughness in comparison with the other substrates tested or the anisotropic nature of the surface finishing. PTFE has a less surface roughness than SS, and a similar morphology (crystal only) can be seen in figure 5*c,e*—this is because PTFE has a low SFE and high receding CA (figure 4). We can safely conclude that CCA mode of evaporation is applicable here.

The contrast between evaporation modes of respiratory fluid on solid substrates not only demonstrates the effect of surface characteristics on drying, but could have significant implications on virus stability in the context of surface transmission and virus detection. Evaporation of water in such a fast timeframe could drastically change the phase and local concentration of virus, protein and salt. Some previous studies suggest that mucin could act as a protective matrix to help virus surviving several days in a completely dried condition [45]. Similar findings were reported for both SARS-CoV and MERS-CoV that could be recovered from inanimate substrates after days, weeks or months [46,47]. The effect of fast transformation with the microenvironment of each respiratory droplet on the structural integrity of a virus is beyond the scope of this work. However, our work shows that appropriate antiviral surface strategies could be developed to inactivate or disrupt virus encapsulated in microdroplet nuclei by modulating the evaporation process on a surface. For example, surface deposited surfactants could be used to influence the Marangoni flow and consequently the evaporation mode. It is equally possible to adjust SFE of a substrate once we find out whether the crystalline or amorphous phase is more effective in disrupting the virus envelope.

### 3.5. Nanomechanical properties of the droplet nuclei

**Figure 7.**
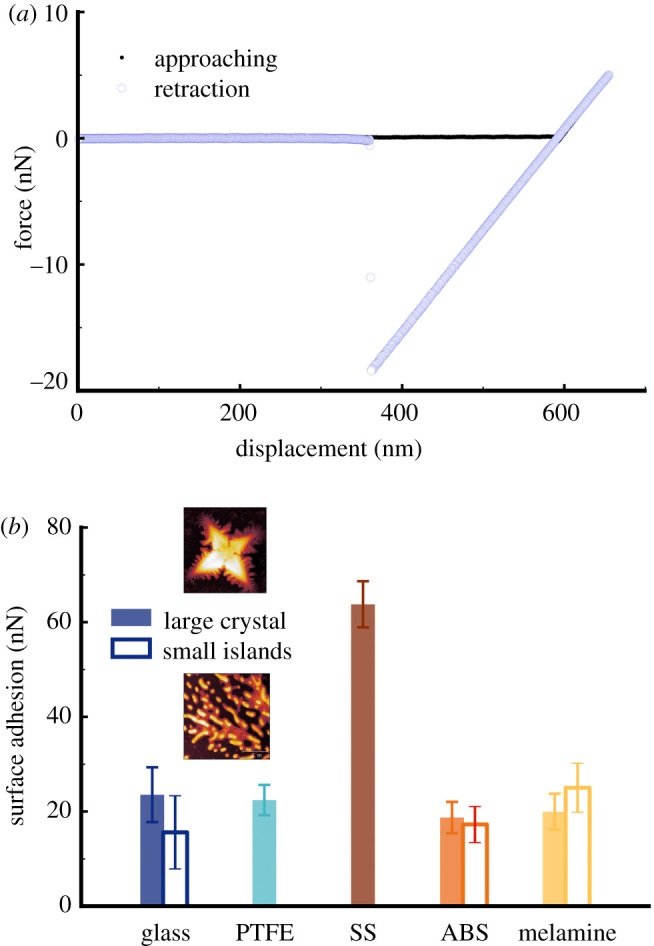
Surface adhesion forces acquired on both the large crystals and the small nuclei area present on all five different substrates. No small islands were observed on PTFE and SS surfaces.

Nanomechanical measurements were performed to further evaluate the proposed ‘core–shell’ structure, during which a nanoscopic tip (radius in the region of 10 nm) made contact with the microdroplet nuclei and retracted subsequently at a given frequency (1 Hz in the present work). Since the experiments were carried out in ambient, capillary force between the AFM tip (silicon nitride) and the nuclei dominates the surface adhesion (recorded as the hysteresis between approaching and retraction curves in figure 7*a*), which can be described as

3.1 $$F_{\mathrm{cap}}=4\pi R\gamma_{L}\cos\theta ,$$

where *R* is the AFM tip radius, *γ*_L_ is the surface tension of water and *θ* is the CA of water on the two surfaces in contact. It is clear that the capillary force is determined by the local SFE of the nuclei that engage with the AFM tip. Used in the past to evaluate the SFE of mineral such as calcite with nanoscopic spatial resolution [48], this method was used in the current study to survey various locations across the microdroplet residue regions, including both the large crystals and the small islands, found on all five inanimate substrates. Equally, AFM-based force spectroscopy could be used to identify any surface heterogeneity since polar chemical moieties would attract water molecules, which consequently result in an increased surface adhesion [49]. At least 100 force curves, with a representative one shown in figure 7*a*, were collected from each point examined to ensure statistical robustness.

Averaged surface adhesion values are presented in figure 7*b*. It shows that very similar magnitudes of surface adhesion were acquired from the microdroplet nuclei deposited on most of the substrates, except SS. The consistent values of surface adhesion on glass, PTFE, ABS and melamine confirm that the SFE of droplet nuclei is very similar, supporting that our proposed ‘core–shell’ model is applicable on these surfaces. The absolute values (around 20 nN) reported agree with a previous study whereby surface adhesion between a silicon AFM cantilever and a glass slide was measured [50] as well a recent work by ourselves concerning surface adhesion on hair fibres in ambient conditions [51]. The nuclei present on SS resulted in a surface adhesion that is approximately three times more than those on the other inanimate substrates, suggesting that they possess high SFE. We speculate that this might be attributed to the exposure of hydrophilic region of mucin on the surface as the result of evaporation of the proteinaceous microdroplets.

**Figure 8.**
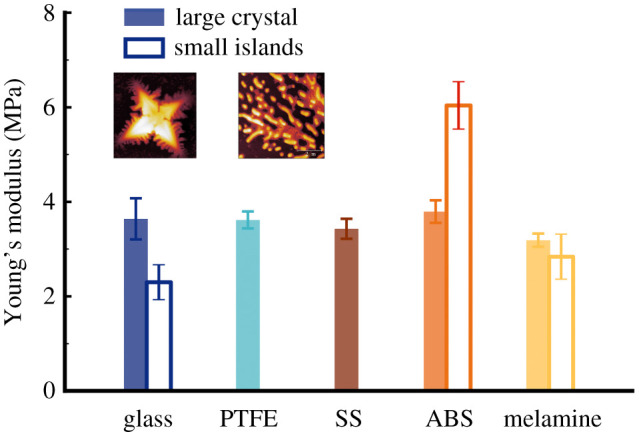
Young's modulus as a function of surface materials receiving droplets. Both large crystals (filled columns) and small nuclei (empty columns) were surveyed.

By indenting the AFM cantilever into the entities present on the solid substrate (droplet nuclei in the present work) for tens of nanometres, it is possible to quantify the viscoelasticity of the nuclei and to evaluate their structural characteristics. Young's moduli of the nuclei, both large crystal and small islands regions, are presented in figure 8. The locations surveyed, across all five substrates, show values of close range (approx. 4 MPa), except the small islands on ABS. The Young's modulus values are consistent with those acquired on polymer film [52], which further supports the proposed structure that mucin molecules present as the shell for the microdroplet nuclei. Although it is not possible to eliminate the influence of the underlying substrate on the Young's modulus, we suggest that the high value acquired from the small islands on ABS is more likely attributed to the variation in the mucin concentration.

## 4. Summary

An advanced inkjet printing method was used to generate microdroplets of respiratory fluid on five common substrates. We show that surface characteristics of the inanimate substrate have a substantial impact on not only the drying kinetics of respiratory fluid microdroplets, but also the properties of the resulting nuclei. The evaporation kinetic of artificial saliva follows CCR mode on substrates with low SFE or great roughness, but CCA mode on those with high SFE. This results in two distinctively different morphologies of the microdroplet nuclei. Atomic force microscopy-based methods, force spectroscopy and nanoindentation were deployed to investigate the nuclei on all five substrates, which could be an invaluable approach in the future studies of respiratory droplets. The nanomechanical measurement results support a core–shell structure of the microdroplet nuclei due to the fast evaporation process of microdroplet, which could have significant implication on the surface viability/transmissibility of viruses as the crystals might preserve the integrity of viruses, or disrupt the virus structure. The work highlights the importance of length scale on the drying of droplets and consequently the possibility of surface transmission of virus-containing small droplets.

## Data accessibility

This article has no additional data.

## References

[1] Leung NHL. 2021 Transmissibility and transmission of respiratory viruses. Nat. Rev. Microbiol. 19, 528-545. (10.1038/s41579-021-00535-6) 33753932 PMC7982882

[2] 2020 COVID-19 transmission—up in the air. Lancet Respir. Med. **8**, 1159. (10.1016/S2213-2600(20)30514-2) 33129420 PMC7598535

[3] van Doremalen N et al. 2020 Aerosol and surface stability of SARS-CoV-2 as compared with SARS-CoV-1. N. Engl. J. Med. **382**, 1564. (10.1056/NEJMc2004973) 32182409 PMC7121658

[4] van Doremalen N et al. 2021 Surface-aerosol stability and pathogenicity of diverse MERS-CoV strains from 2012–2018. *bioRxiv*. (10.1101/2021.02.11.429193)

[5] Tang JW, Marr LC, Li Y, Dancer SJ. 2021 Covid-19 has redefined airborne transmission. BMJ **373**, N913. (10.1136/bmj.n913) 33853842

[6] Goldman E. 2020 Exaggerated risk of transmission of COVID-19 by fomites. Lancet Infect. Dis. **20**, 892. (10.1016/S1473-3099(20)30561-2) 32628907 PMC7333993

[7] Greenhalgh T, Jimenez JL, Prather KA, Tufekci Z, Fisman D, Schooley R. 2021 Ten scientific reasons in support of airborne transmission of SARS-CoV-2. Lancet **397**, 1603. (10.1016/S0140-6736(21)00869-2) 33865497 PMC8049599

[8] Goldman E. 2021 SARS wars: the fomites strike back. Appl. Environ. Microbiol. **87**, e00653-21. (10.1128/AEM.00653-21) PMC8316018 33931423

[9] Onakpoya IJ, Heneghan CJ, Spencer EA, Brassey J, Plüddemann A, Evans DH, Conly JA, Jefferson T. 2021 SARS-CoV-2 and the role of fomite transmission: a systematic review version 1; peer review: 2 approved with reservations. F1000Research **10**, 233. (10.12688/f1000research.51590.1) 34136133 PMC8176266

[10] Ijaz MK, Nims RW, McKinney J. 2021 Indirect transmission of severe acute respiratory syndrome coronavirus virus 2 (SARS-CoV-2): what do we know and what do we not know? Infect. Control Hosp. Epidemiol. 1-2. (10.1017/ice.2021.57) PMC7943946 33557961

[11] Tang JW et al. 2021 Dismantling myths on the airborne transmission of severe acute respiratory syndrome coronavirus-2 (SARS-CoV-2). J. Hosp. Infect. **110**, 89. (10.1016/j.jhin.2020.12.022) 33453351 PMC7805396

[12] Bake B, Larsson P, Ljungkvist G, Ljungstrom E, Olin AC. 2019 Exhaled particles and small airways. Respir. Res. **20**, 8. (10.1186/s12931-019-0970-9) 30634967 PMC6330423

[13] Morawska LJGR, Johnson GR, Ristovski ZD, Hargreaves M, Mengersen K, Corbett S, Chao CYH, Li Y, Katoshevski D. 2009 Size distribution and sites of origin of droplets expelled from the human respiratory tract during expiratory activities. J. Aerosol Sci. **40**, 256. (10.1016/j.jaerosci.2008.11.002) PMC7126899 32287373

[14] Fabian P, McDevitt JJ, DeHaan WH, Fung RO, Cowling BJ, Chan KH, Leung GM, Milton DK. 2008 Influenza virus in human exhaled breath: an observational study. PLoS ONE **3**, e2691. (10.1371/journal.pone.0002691) 18628983 PMC2442192

[15] Papineni RS, Rosenthal FS. 1997 The size distribution of droplets in the exhaled breath of healthy human subjects. J. Aerosol Med. **10**, 105. (10.1089/jam.1997.10.105) 10168531

[16] Tang JW, Li Y, Eames I, Chan PK, Ridgway GL. 2006 Factors involved in the aerosol transmission of infection and control of ventilation in healthcare premises. J. Hosp. Infect. **64**, 100. (10.1016/j.jhin.2006.05.022) 16916564 PMC7114857

[17] Edwards DA, Man JC, Brand P, Katstra JP, Sommerer K, Stone HA, Nardell E, Scheuch G. 2004 Inhaling to mitigate exhaled bioaerosols. Proc. Natl Acad. Sci. USA **101**, 17383. (10.1073/pnas.0408159101) 15583121 PMC536048

[18] Cazabat AM, Guéna G. 2010 Evaporation of macroscopic sessile droplets. Soft Matter **6**, 2591. (10.1039/b924477h)

[19] Stauber JM, Wilson SK, Duffy BR, Sefiane K. 2015 On the lifetimes of evaporating droplets with related initial and receding contact angles. Phys. Fluids **27**, 122101. (10.1063/1.4935232)

[20] Fedorenko A, Grinberg M, Orevi T, Kashtan N. 2020 Survival of the enveloped bacteriophage Phi6 (a surrogate for SARS-CoV-2) in evaporated saliva microdroplets deposited on glass surfaces. Sci. Rep. **10**, 22419. (10.1038/s41598-020-79625-z) 33376251 PMC7772334

[21] Di Novo NG, Carotenuto AR, Mensitieri G, Fraldi M, Pugno NM. 2021 Modeling of virus survival time in respiratory droplets on surfaces: a new rational approach for antivirus strategies. Front. Mater. **8**, 631723. (10.3389/fmats.2021.631723)

[22] Vejerano EP, Marr LC. 2018 Physico-chemical characteristics of evaporating respiratory fluid droplets. J. R. Soc. Interface **15**, 20170939. (10.1098/rsif.2017.0939) 29491178 PMC5832737

[23] Marr LC, Tang JW, Van Mullekom JV, Lakdawala SS. 2019 Mechanistic insights into the effect of humidity on airborne influenza virus survival, transmission and incidence. J. R. Soc. Interface **16**, 20180298. (10.1098/rsif.2018.0298) 30958176 PMC6364647

[24] World Health Organization. 2020 Surface sampling of coronavirus disease (COVID-19): a practical ‘how to’ protocol for health care and public health professionals.

[25] Malamud D, Abrams WR, Barber CA, Weissman D, Rehtanz M, Golub E. 2011 Antiviral activities in human saliva. Adv. Dent. Res. **23**, 34. (10.1177/0022034511399282) 21441478 PMC3144043

[26] White MR, Helmerhorst EJ, Ligtenberg A, Karpel M, Tecle T, Siqueira WL, Oppenheim FG, Hartshorn KL. 2009 Multiple components contribute to ability of saliva to inhibit influenza viruses. Oral Microbiol. Immunol. **24**, 18. (10.1111/j.1399-302X.2008.00468.x) 19121065 PMC2848456

[27] Shugars DC. 1999 Endogenous mucosal antiviral factors of the oral cavity. J. Infect. Dis. **179**, S431. (10.1086/314799) 10099113

[28] Diaz-Arnold AM, Marek CA. 2002 The impact of saliva on patient care: a literature review. J. Prosthet. Dent. **88**, 337. (10.1067/mpr.2002.128176) 12426506

[29] Dodds MW, Johnson DA, Yeh CK. 2005 Health benefits of saliva: a review. J. Dent. **33**, 223-233. (10.1016/j.jdent.2004.10.009) 15725522

[30] Humphrey SP, Williamson RT. 2001 A review of saliva: normal composition, flow, and function. J. Prosthet. Dent. **85**, 162. (10.1067/mpr.2001.113778) 11208206

[31] Aps JK, Martens LC. 2005 Review: the physiology of saliva and transfer of drugs into saliva. Forensic Sci. Int. **150**, 119. (10.1016/j.forsciint.2004.10.026) 15944052

[32] Veerman EC, van den Keybus PA, Vissink A, Nieuw Amerongen AV. 1996 Human glandular salivas: their separate collection and analysis. Eur. J. Oral Sci. **104**, 346. (10.1111/j.1600-0722.1996.tb00090.x) 8930581

[33] Wong L, Sissons C. 2001 A comparison of human dental plaque microcosm biofilms grown in an undefined medium and a chemically defined artificial saliva. Arch. Oral Biol. **46**, 477. (10.1016/S0003-9969(01)00016-4) 11311195

[34] Owens DK, Wendt RC. 1969 Estimation of the surface free energy of polymers. J. Appl. Polym. **13**, 1741. (10.1002/app.1969.070130815)

[35] Good RJ, Girifalco LA. 1960 A theory for estimation of surface and interfacial energies. III. Estimation of surface energies of solids from contact angle data. J. Phys. Chem. **64**, 561-565. (10.1021/j100834a012)

[36] Picknett RG, Bexon R. 1977 The evaporation of sessile or pendant drops in still air. J. Colloid Interface Sci. **61**, 336. (10.1016/0021-9797(77)90396-4)

[37] Stauber JM, Wilson SK, Duffy BR, Sefiane K. 2015 Evaporation of droplets on strongly hydrophobic substrates. Langmuir **31**, 3653. (10.1021/acs.langmuir.5b00286) 25747121

[38] Talbot EL, Berson A, Brown PS, Bain CD. 2012 Evaporation of picoliter droplets on surfaces with a range of wettabilities and thermal conductivities. Phys. Rev. E **85**, 061604. (10.1103/PhysRevE.85.061604) 23005106

[39] Bonn D, Eggers J, Indekeu J, Meunier J, Rolley E. 2009 Wetting and spreading. Rev. Mod. Phys. **81**, 739. (10.1103/RevModPhys.81.739)

[40] Sheng YJ, Jiang S, Tsao HK. 2007 Effects of geometrical characteristics of surface roughness on droplet wetting. J. Chem. Phys. **127**, 234704. (10.1063/1.2804425) 18154406

[41] Ávila-Sierra A, Zhang ZJ, Fryer PJ. 2021 Effect of surface roughness and temperature on stainless steel–whey protein interfacial interactions under pasteurisation conditions. J. Food Eng. **301**, 110542. (10.1016/j.jfoodeng.2021.110542)

[42] Lieber C, Melekidis S, Koch R, Bauer HJ. 2021 Insights into the evaporation characteristics of saliva droplets and aerosols: levitation experiments and numerical modeling. J. Aerosol. Sci. **154**, 105760. (10.1016/j.jaerosci.2021.105760) 33518792 PMC7826107

[43] Deegan RD, Bakajin O, Dupont TF, Huber G, Nagel SR, Witten TA. 1997 Capillary flow as the cause of ring stains from dried liquid drops. Nature **389**, 827. (10.1038/39827)

[44] Hu H, Larson RG. 2006 Marangoni effect reverses coffee-ring depositions. J. Phys. Chem. B **110**, 7090. (10.1021/jp0609232) 16599468

[45] Thomas Y, Vogel G, Wunderli W, Suter P, Witschi M, Koch D, Tapparel C, Kaiser L. 2008 Survival of influenza virus on banknotes. Appl. Environ. Microbiol. **74**, 3002. (10.1128/AEM.00076-08) 18359825 PMC2394922

[46] Otter JA, Donskey C, Yezli S, Douthwaite S, Goldenberg SD, Weber DJ. 2016 Transmission of SARS and MERS coronaviruses and influenza virus in healthcare settings: the possible role of dry surface contamination. J. Hosp. Infect. **92**, 235. (10.1016/j.jhin.2015.08.027) 26597631 PMC7114921

[47] Kampf G, Todt D, Pfaender S, Steinmann E. 2020 Persistence of coronaviruses on inanimate surfaces and their inactivation with biocidal agents. J. Hosp. Infect. **104**, 246. (10.1016/j.jhin.2020.01.022) 32035997 PMC7132493

[48] Sauerer B, Stukan M, Abdallah W, Derkani MH, Fedorov M, Buiting J, Zhang ZJ. 2016 Quantifying mineral surface energy by scanning force microscopy. J. Colloid Interface Sci. **472**, 237. (10.1016/j.jcis.2016.03.049) 27054773

[49] Butt HJ, Cappella B, Kappl M. 2005 Force measurements with the atomic force microscope: technique, interpretation and applications. Surf. Sci. Rep. **59**, 1-152. (10.1016/j.surfrep.2005.08.003)

[50] Jones R, Pollock HM, Cleaver JAS, Hodges CS. 2002 Adhesion forces between glass and silicon surfaces in air studied by AFM: effects of relative humidity, particle size, roughness, and surface treatment. Langmuir **18**, 8045. (10.1021/la0259196)

[51] Labarre L, Squillace O, Liu Y, Fryer PJ, Kaur P, Whitaker S, Marsh JM, Zhang ZJ. Submitted. Hair surface interactions against different chemical functional groups as a function of environment and hair condition. 10.1111/ics.12834 PMC10946710 36683407

[52] Sun Y, Akhremitchev B, Walker GC. 2004 Using the adhesive interaction between atomic force microscopy tips and polymer surfaces to measure the elastic modulus of compliant samples. Langmuir **20**, 5837. (10.1021/la036461q) 16459598

